# Impact of Toceranib/Piroxicam/Cyclophosphamide Maintenance Therapy on Outcome of Dogs with Appendicular Osteosarcoma following Amputation and Carboplatin Chemotherapy: A Multi-Institutional Study

**DOI:** 10.1371/journal.pone.0124889

**Published:** 2015-04-29

**Authors:** Cheryl A. London, Heather L. Gardner, Tamra Mathie, Nicole Stingle, Roberta Portela, Michael L. Pennell, Craig A. Clifford, Mona P. Rosenberg, David M. Vail, Laurel E. Williams, Kim L. Cronin, Heather Wilson-Robles, Antonella Borgatti, Carolyn J. Henry, Dennis B. Bailey, Jennifer Locke, Nicole C. Northrup, Martin Crawford-Jakubiak, Virginia L. Gill, Mary K. Klein, David M. Ruslander, Doug H. Thamm, Brenda Phillips, Gerald Post

**Affiliations:** 1 Departments of Veterinary Biosciences and Veterinary Clinical Sciences, College of Veterinary Medicine, The Ohio State University, Columbus, Ohio, United States of America; 2 Department of Veterinary Clinical Medicine, University of Illinois at Urbana-Champaign, College of Veterinary Medicine, Champaign, Illinois, United States of America; 3 Division of Biostatistics, College of Public Health, The Ohio State University, Columbus, Ohio, United States of America; 4 Hope Veterinary Specialists, Malvern, Pennsylvania, United States of America; 5 Veterinary Cancer Group, Tustin, California, United States of America; 6 Department of Medical Sciences, School of Veterinary Medicine, University of Wisconsin, Madison, Madison, Wisconsin, United States of America; 7 Department of Clinical Sciences, College of Veterinary Medicine, North Carolina State University, Raleigh, North Carolina, United States of America; 8 New England Veterinary Oncology Group, Waltham, Massachusetts, United States of America; 9 Department of Small Animal Clinical Sciences, College of Veterinary Medicine, Texas A&M University, College Station, Texas, United States of America; 10 Department of Veterinary Clinical Sciences, University of Minnesota College of Veterinary Medicine, St. Paul, Minnesota, United States of America; 11 Department of Veterinary Medicine and Surgery, College of Veterinary Medicine, University of Missouri, Columbia, Missouri, United States of America; 12 Oradell Animal Hospital, Paramus, New Jersey, United States of America; 13 Southeast Veterinary Oncology and Medicine, Orange Park, Florida, United States of America; 14 Department of Small Animal Medicine and Surgery, University of Georgia, College of Veterinary Medicine, Athens, Georgia, United States of America; 15 Sage Centers for Veterinary Specialty and Emergency Care, Concord, California, United States of America; 16 VCA Katonah Bedford Veterinary Center, Bedford Hill, New York, United States of America; 17 Southwest Veterinary Oncology, Tucson, Arizona, United States of America; 18 Veterinary Specialty Hospital of the Carolinas, Cary, North Carolina, United States of America; 19 Department of Clinical Sciences, College of Veterinary Medicine and Biomedical Sciences, Colorado State University, Fort Collins, Colorado, United States of America; 20 Veterinary Specialty Hospital of San Diego, San Diego, California, United States of America; 21 The Veterinary Cancer Center, Norwalk, Connecticut, United States of America; Institut Albert Bonniot-INSERMU823, FRANCE

## Abstract

**Background:**

We hypothesized that the addition of toceranib to metronomic cyclophosphamide/piroxicam therapy would significantly improve disease-free interval (DFI) and overall survival (OS) in dogs with appendicular osteosarcoma (OSA) following amputation and carboplatin chemotherapy.

**Methods and Findings:**

This was a randomized, prospective clinical trial in which dogs with OSA free of gross metastatic disease (n = 126) received carboplatin chemotherapy (4 doses) following amputation. On study entry, dogs were randomized to receive piroxicam/cyclophosphamide with or without toceranib (n = 63 each) after completing chemotherapy. Patient demographics were not significantly different between both groups. During or immediately following carboplatin chemotherapy, 32 dogs (n = 13 toceranib; n = 19 control) developed metastatic disease, and 13 dogs left the study due to other medical conditions or owner preference. Following carboplatin chemotherapy, 81 dogs (n = 46 toceranib; n = 35 control) received the metronomic treatment; 35 dogs (n = 20 toceranib; n = 15 control) developed metastatic disease during the maintenance therapy, and 26 dogs left the study due to other medical conditions or owner preference. Nine toceranib-treated and 11 control dogs completed the study without evidence of metastatic disease 1-year following amputation. Toceranib-treated dogs experienced more episodes of diarrhea, neutropenia and weight loss than control dogs, although these toxicities were low-grade and typically resolved with supportive care. More toceranib-treated dogs (n = 8) were removed from the study for therapy-associated adverse events compared to control dogs (n = 1). The median DFI for control and toceranib treated dogs was 215 and 233 days, respectively (p = 0.274); the median OS for control and toceranib treated dogs was 242 and 318 days, respectively (p = 0.08). The one year survival rate for control dogs was 35% compared to 38% for dogs receiving toceranib.

**Conclusions:**

The addition of toceranib to metronomic piroxicam/cyclophosphamide therapy following amputation and carboplatin chemotherapy did not improve median DFI, OS or the 1-year survival rate in dogs with OSA.

## Introduction

Osteosarcoma (OSA) is the most common primary bone tumor in dogs, comprising up to 85% of all reported bone neoplasia [1]. Median survival times approach 4–5 months with amputation alone, and adjuvant chemotherapy improves median survival times to 8–12 months[2]. Despite the use of various chemotherapy protocols and novel treatment approaches, clinically meaningful improvements in survival have not been achieved and 90% of dogs die of metastatic disease within 2 years after treatment [3–8].

Given that most dogs with OSA possess microscopic metastatic disease at the time of presentation, a goal of therapeutic development has been to influence factors in the tumor microenvironment that are critical for outgrowth of the metastatic tumor cells. In particular, efforts have been directed at modulating immune responses and altering tumor access to critical elements, such as blood supply as an alternative to the standard cytotoxic approaches to treatment. In veterinary medicine, metronomic chemotherapy typically consists of piroxicam and low-dose cyclophosphamide and several publications have demonstrated modulation of both the tumor microenvironment and immune response with this approach [9–11]. In both veterinary and human medicine, metronomic cyclophosphamide has been shown to downregulate CD4+ Tregs [11,12]. Metronomic cyclophosphamide administered at 15 mg/m2/day has also been associated with decreased tumor microvessel density in dogs with soft tissue sarcoma [11]. In addition, metronomic cyclophosphamide has been shown to decrease mobilization and viability of circulating endothelial precursors (CEPs) [13,14].

While NSAIDs are not included in most human metronomic chemotherapy protocols, piroxicam has been frequently used in veterinary medicine as it has been hypothesized that COX-2 expression in CEPs is important for their survival. As such COX-2 inhibition may decrease the ability of CEPs to survive and proliferate in the tumor microenvironment [15]. The combination of metronomic cyclophosphamide with piroxicam in mice with canine melanoma xenografts resulted in decreased tumor microvessel density, decreased VEGF secretion and increased TSP-1 secretion, supporting the concurrent use of metronomic chemotherapy and NSAIDs [16].

With respect to tumor angiogenesis, vascular endothelial growth factor (VEGF) and its receptor (VEGFR) are known to play critical roles in this process. In endothelial cells activation of the VEGFR stimulates multiple signaling pathways that promote endothelial cell survival, increased vascular permeability and mobilization of CEPs. Tumor cells can drive the migration of VEGFR2 expressing CEPs from the bone marrow to the tumor microenvironment through the production of VEGF and anti-VEGF/VEGFR therapy has been shown to decrease survival signaling and mobilization of CEPs to the site of tumor growth [17–20]. VEGF is detectable in both human and canine OSA, and has been associated with increased malignant potential and poor prognosis [21–25].

In human cancer therapy there are several approved inhibitors of the VEGF/VEGFR signaling axis, including the small molecules sunitinib, sorafenib, and pazopanib and the monoclonal antibody bevacizumab. While these drugs exhibit significant activity in mouse models of disease, their effects on angiogenesis and tumor progression in humans have been questionable [26–29]. With respect to OSA, several multi-targeted VEGF inhibitors have been evaluated in murine xenograft models of OSA and have demonstrated antitumor activity [30,31]. In addition, cediranib combined with gefitinib demonstrated antitumor activity and changes in VEGF and VEGFR2 levels in patients with solid tumors, including one with OSA[32]. Toceranib phosphate (toceranib; Palladia) is a multi-targeted small molecule inhibitor of several receptor tyrosine kinases (RTKs) including VEGFR, PDGFR, KIT, and FLT-3 that was developed to be an orally bioavailable anti-angiogenic agent [33–35]. Although toceranib was approved for the treatment of canine mast cell tumors by virtue of its inhibition of KIT, activity against a wide variety of tumors has been demonstrated in dogs, likely due to dysregulation of other RTKs inhibited by toceranib [33,36]. Previously published data demonstrate that doses of 2.4–2.75 mg/kg of toceranib given every other day result in statistically significant increases in plasma VEGF, a surrogate biomarker of VEGFR2 inhibition [37]. As such, the doses of toceranib currently used to treat dogs for a variety of tumors would be expected to have a similar effect.

In the setting of metastatic OSA, a retrospective study showed that approximately 48% of dogs experienced clinical benefit (primarily consisting of stable disease) following toceranib therapy [38]. In this study, many of the dogs were also treated with metronomic cyclophosphamide and/or an NSAID in conjunction with toceranib. More recently, toceranib has been shown to downregulate levels of circulating regulatory T cells in dogs with cancer, suggesting that some of its effects on tumors may be exerted through immunomodulation [39].

Given the potential complementary effects of metronomic chemotherapy and VEGF/VEGFR inhibitors, they have been combined in human medicine with variable outcomes [26,40–42]. However, in women with advanced breast cancer clinical benefit was documented in a substantial number of patients (63.6–68%) using combinations of bevacizumb and cyclophosphamide with capecitabine or methotrexate [26,42]. These data suggest that administration of continuous low-dose metronomic chemotherapy with anti-angiogenic targeted therapies may represent a valid strategy to address microscopic metastatic disease. Therefore, the purpose of this clinical trial was to evaluate the impact of toceranib phosphate combined with metronomic cyclophosphamide and piroxicam on disease free interval (DFI) and overall survival (OS) in dogs with appendicular OSA following amputation and carboplatin chemotherapy.

## Materials and Methods

### Eligibility and Ethics Statement

The Clinical Research and Advising Committee at the College of Veterinary Medicine at The Ohio State University and the Institutional Animal Care and Use Committee (IACUC) at The Ohio State University approved this study. IACUC approval was also obtained at the University of Wisconsin-Madison, North Carolina State University, Texas A&M University, University of Minnesota, University of Missouri, University of Georgia, Colorado State University. The private specialty practices that participated in this clinical trial do not require IACUC approval; participation in the clinical trial was at the discretion of the Veterinary Medical Oncologist designated as principle investigator at these study sites (Hope Veterinary Specialists, Malvern, PA; Veterinary Cancer Group, Tustin, CA,; New England Veterinary Oncology Group, Waltham, MA; Oradell Animal Hospital, Paramus, NJ; Southeast Veterinary Oncology and Medicine, Orange Park, FL; Sage Centers for Veterinary Specialty and Emergency Care, Concord, CA; VCA Katonah Bedford Veterinary Center, Bedford Hill, NY; Southwest Veterinary Oncology, Tucson, AZ; Veterinary Specialty Hospital of the Carolinas, Cary, NC; Veterinary Specialty Hospital, San Diego, CA; The Veterinary Cancer Center, Norwalk, CT). Informed consent was obtained from all owners prior to study entry.

Dogs free of gross metastatic disease with histologically confirmed appendicular osteosarcoma that had undergone amputation were considered for enrollment. Only appendicular sites involving long bones were considered for enrollment. Prior to enrollment, dogs underwent diagnostic tests including thoracic radiographs, complete blood count (CBC), serum biochemistry profile and urinalysis. Dogs were excluded if they received prior treatment with radiation therapy or chemotherapy, or had received non-steroidal anti-inflammatory (NSAID) drugs within 72 hours of beginning carboplatin chemotherapy.

### Study Design

A total of 126 dogs with histologically confirmed appendicular osteosarcoma without evidence of gross metastatic disease were enrolled in this study following amputation. A computer generated randomization table was established prior to study initiation and study group assignment followed this table as patients were enrolled. Upon enrollment, dogs were randomized to receive piroxicam/cyclophosphamide with or without toceranib (provided by Zoetis, Florham Park, NJ) after completing carboplatin chemotherapy. All dogs began treatment with 4 cycles of single agent carboplatin (300 mg/m2 IV) every three weeks within 14 days of amputation. Prior to the fourth carboplatin treatment dogs were re-evaluated with thoracic radiographs. Dogs without evidence of pulmonary metastatic disease began oral maintenance therapy with piroxicam at 0.3 mg/kg PO EOD and cyclophosphamide at 10 mg/m2 PO EOD (alternating day of dosing with toceranib) with or without toceranib at 2.75 mg/kg PO every other day (EOD); oral therapy was initiated 3 weeks after completion of the fourth carboplatin treatment. All dogs received 0.5 mg/kg PO famotidine every 12 hours at the start of oral maintenance therapy. Chlorambucil was administered at 5 mg/m2 PO EOD in place of cyclophosphamide in the event of sterile hemorrhagic cystitis. Dogs were evaluated 2 weeks after starting oral maintenance therapy, then once every 4 weeks thereafter for the next 8 months or until progressive disease was noted. CBC and biochemistry profile were performed every 4 weeks; urinalysis was obtained every 16 weeks after starting oral maintenance therapy. Restaging with thoracic radiographs was performed every 8 weeks.

### Drug Products and Concomitant Medications

Cyclophosphamide and piroxicam were compounded by the Apothecary Shoppe (now Avella Specialty Pharmacy, Columbus, OH) and mailed directly to owners. The Apothecary Shoppe is certified by the Pharmacy Compounding Accreditation Board (PCAB), and was used to decrease the likelihood of inaccurate dosing. Chlorambucil was compounded for the 8 dogs that developed cystitis/gastroenteritis on the cyclophosphamide by Diamondback Drugs (Phoenix Arizona). Toceranib was provided by Pfizer Animal Heath (now Zoetis) in 10 mg, 15 mg and 50 mg size tablets.

Adverse events were recorded and graded using the VCOG-CTCAE [43]. Concomitant medications to prevent and/or treat drug related toxicities were used at the discretion of the attending clinician and included the following: antibiotics (ciprofloxacin, cefazolin, cephalexin, amoxicillin clavulanic acid, trimethoprim-sulfamethoxazole, enrofloxacin, marbofloxacin), anti-emetics (ondansetron, maropitant, metoclopramide), gastrointestinal protectants (omeprazole, famotidine, sucralfate, pantoprazole, misoprostol, ranitidine), anti-diarrheal (loperamide, metronidazole, Pepto-Bismol, probiotics), and pain (tramadol, gabapentin, buprenorphine, fentanyl), proteinuria/hypertension (benazepril, enalapril), elevated liver transaminases (Denamarin, SAMe).

### Statistical Analysis

Historical control groups were taken from two previous studies of 48 and 155 dogs with osteosarcoma treated with amputation and carboplatin chemotherapy to perform sample size calculations for the study. The reported 1-year survival rate in these two populations was 35% [3,7]. Assuming 80% power and 95% confidence, 47 dogs in each group were required to demonstrate a 50% increase in the 1-year survival rate reported in the historical control population. Due to the high attrition rate, 32 additional dogs (63 dogs total per group) were enrolled for a total of 126 dogs. Patient characteristics were summarized by treatment group (mean ± standard deviation (SD) for continuous variables and frequency (%) for categorical variables). The period between the date of amputation and development of detectable metastatic disease was defined as the disease free interval (DFI). Dogs were censored from the DFI analysis if they did not have documented metastatic disease at the time of death or last follow-up. Necropsy examination was not required, however dogs with radiographic, cytologic/histopathologic evidence of disease progression were included in the DFI analysis. Overall survival (OS) was defined as the time from amputation to the date of death or euthanasia. Dogs were censored from the survival analysis if they were alive at the time of last follow-up or were lost to follow-up. Kaplan-Meier curves were used to describe DFI and OS for each group and Cox regression was used to test for a difference in DFI or OS adjusting for characteristics found to differ by group at baseline. OS was chosen instead of disease-specific survival to avoid under-reporting of disease-specific deaths. Plots of Schoenfeld Residuals [44,45] and tests of an interaction with time (log-transformed) were used to determine if the proportional hazards (PH) assumption of Cox regression was violated. If PH was violated, the interaction with time was retained and hazard ratios were estimated at four time points: 100 days (roughly the time at which maintenance therapy began), time at which the hazards of the two groups converged, one time point halfway between 100 days and time of convergence, and a final time point halfway between the time of convergence and the maximum follow-up time. Two types of analyses were performed for DFI: an intent-to-treat analysis and an adherence analysis. Dogs in both analyses were censored if there was no evidence of metastatic disease. In the intent-to-treat analysis, dogs free of metastatic disease were censored at the last known time alive without metastatic disease. For the adherence analysis, dogs that left the study prior to the development of metastatic disease due to owner preference or another medical condition were censored at the time they withdrew from the study. The remaining dogs in the adherence analysis were censored at the last known time without metastatic disease. An adherence analysis was not performed for OS since many patients withdrew due to medical issues that put them at a greater risk of death than patients who remained in the study; had these patients been included in the analysis, the independent censoring assumption of the Kaplan-Meier estimator and Cox regression would have been violated. Survival outcomes were also compared by tumor location (proximal humerus versus all other locations) to assess whether location impacted outcome between treatment groups. For adverse events that were experienced by at least five patients and at least two per treatment group, event rates were compared across treatment groups using Poisson regression with robust standard errors estimated using Generalized Estimating Equations (GEE). Adverse events occurring in only one treatment group were rare, and only those common enough to be compared statistically were included in this analysis. Survival analyses were performed using Intercooled Stata 11 (StataCorp, College Station, TX). Poisson regression was performed using PROC GENMOD in SAS version 9.2 (SAS Inc., Cary, NC).

## Results

### Patient Demographics

This was a multi-institutional study, with a total of 126 dogs (63 toceranib-treated, 63 control) enrolled from September 2010 through August 2012. Patients in the control group were heavier and more likely to be male (Table 1). Thus, adjustments were made for patient weight and gender (male, female ignoring castrated/spayed status) in the Cox regression analyses.

**Table 1:** Distribution of patient characteristics.

Variable		Toceranib (n = 63)	Control (n = 63)	Total (n = 126)
**Age (yrs)**		8.4 ± 2.7	8.0 ± 2.6	8.2 ± 2.6
**Weight (kg)**		35.9 ± 10.4	40.8 ± 11.8	38.3 ± 11.3
**Gender**	Female	0 (0%)	1 (1.6%)	1 (0.8%)
	Female Spayed	36 (57.1%)	29 (46.0%)	65 (51.6%)
	Male	1 (1.6%)	1 (1.6%)	2 (1.6%)
	Male Castrated	26 (41.3%)	32 (50.8%)	58 (46.0%)
**Breed**	Pure Bred	13 (20.6%)	11 (17.5%)	24 (19.1%)
	Mixed Breed	50 (79.4%)	52 (82.5%)	102 (80.9%)
**Tumor Location**	Proximal Humerus	16 (25.4%)	15 (23.8%)	31 (24.6%)
	Other	47 (74.6%)	48 (76.2%)	95 (75.4%)

### Dosage and Drug Modifications

The starting dose of carboplatin for all dogs was 300 mg/m2. The median dose of carboplatin administered during the study was 300 mg/m2. Nineteen dogs had dose adjustments (range: 250–290 mg/m2). More than one dose adjustment occurred in 4 dogs. Carboplatin dose-delays due to adverse events occurred in 33 dogs. The median dose of toceranib administered was 2.73 mg/kg EOD. Twenty-seven dogs had toceranib dose-reductions. In addition, temporary toceranib discontinuation occurred in 10 dogs due to adverse events, prior to re-instituting therapy. Drug discontinuation generally lasted 1 week. Cyclophosphamide-induced cystitis resulted in 7 dogs switching from cyclophosphamide to chlorambucil at 5 mg/m2 EOD. Chlorambucil was substituted for cyclophosphamide in 1 dog due to gastrointestinal adverse events.

### Patient Outcome

Thirty-two dogs (25%) developed progressive disease prior to beginning oral therapy at week 14, of which 12 dogs developed metastatic lesions outside of the pulmonary parenchyma. This is consistent with previous reports of carboplatin chemotherapy in dogs with appendicular OSA. Thirteen dogs withdrew prior to starting oral maintenance therapy due to owner non-compliance or other unrelated medical conditions. Of the 126 dogs entered into the study, 81 dogs (64%) received the metronomic treatment. Of the 46 dogs that received oral therapy with toceranib, 9 dogs completed the study protocol, 20 dogs withdrew due to the development of metastatic disease, and 17 withdrew due to owner non-compliance or other unrelated medical conditions. Of the 35 dogs in the control group that received oral therapy without toceranib, 11 dogs completed the study protocol, 15 dogs withdrew due to the development of metastatic disease, and 9 withdrew due to owner non-compliance or other unrelated medical conditions.

**Fig 1:**
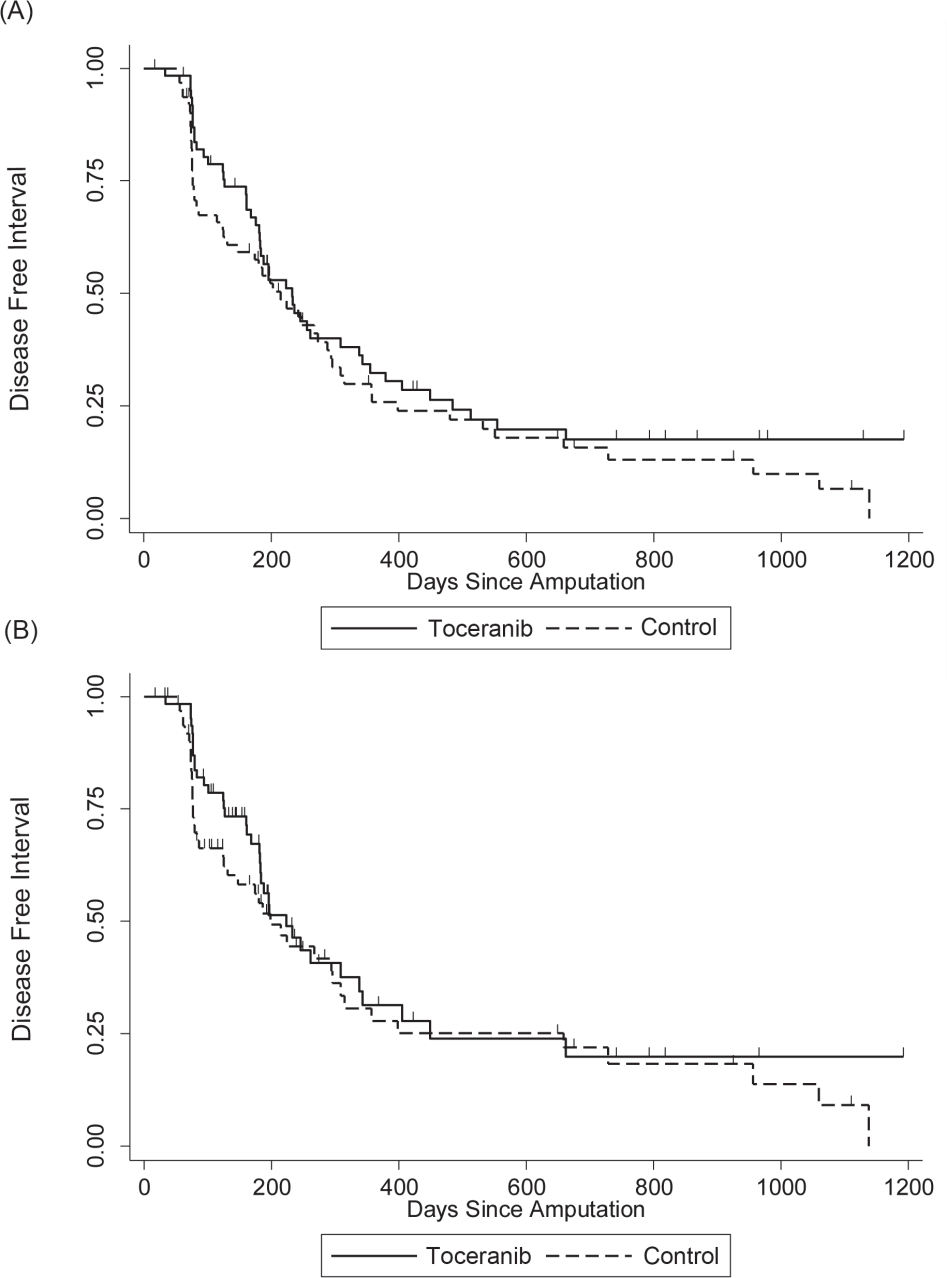
Disease Free Interval. Kaplan-Meier disease-free interval (DFI) curves comparing dogs treated with toceranib to control dogs. Hash marks denote censored observations; n = 63 toceranib-treated dogs; n = 63 control dogs. (A) Intent-to-treat analysis (p = 0.274). (B) Adherence analysis (p = 0.3).

**Fig 2:**
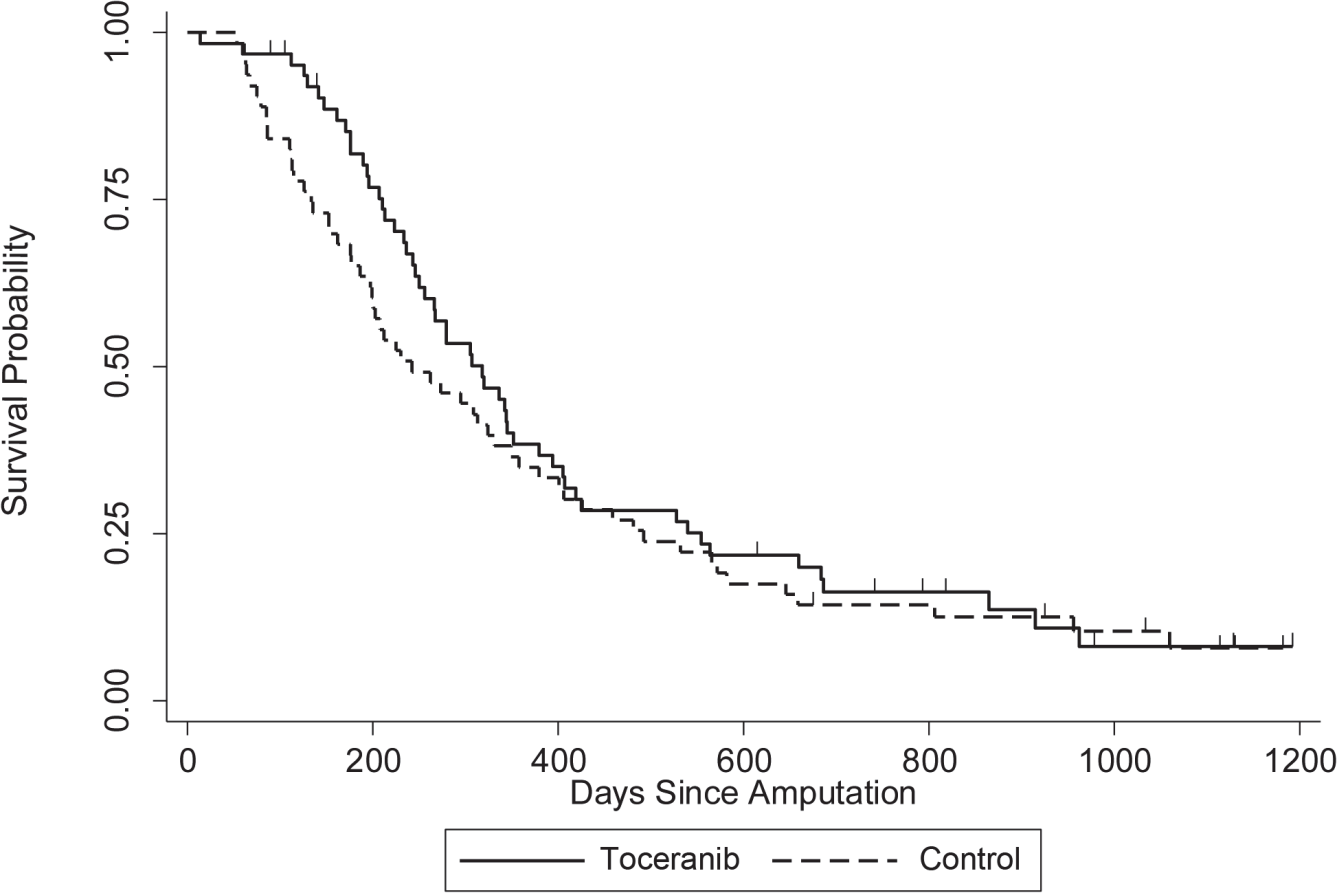
Overall Survival. Kaplain-Meier overall survival (OS) curves comparing toceranib-treated dogs with control dogs. Hash marks denote censored observations; n = 63 toceranib-treated dogs; n = 63 control dogs (p = 0.08).

The median DFI reported in the intent-to-treat analysis was 233 days for toceranib-treated dogs and 215 days for control dogs (p = 0.274, Fig 1A, Tables 2 and 3). The median DFI reported in the adherence analysis was 223 days for toceranib-treated dogs and 198 days for control dogs (p = 0.3, Fig 1B, Tables 2 and 3). The median OS was 318 days and 242 days for toceranib-treated and control dogs, respectively (p = 0.08) (Fig 2, Tables 2 and 3). DFI was similar across treatment groups, however there was a time-dependent difference in short-term mortality risk (Fig 2 and Table 3). Initially, the mortality hazard was greater among control patients, but at approximately 400 days the risks converged and were not significantly different at one year (p = 0.961) and two years (p = 0.316). The 1- and 2-year survival rates of toceranib treated group were not significantly different from those of the control group (p = 0.963 and 0.325, respectively) (Table 2). Median DFI (182 days, p = 0.281) and OS (243 days, p = 0.28) were shorter among patients whose cancer was located in the proximal humerus, but this difference was not significant (Table 4). In addition, proximal humeral location did not affect the differences between treatment groups (p > 0.28 for each survival outcome).

**Table 2:** Median Disease Free Interval (DFI), Overall Survival (OS) and survival proportions by treatment group.

Outcome			Survival Proportions		
Analysis	Group	Median (days)	1 year	2 years	3 years
**DFI ITT**	Toceranib	233	0.323	0.176	0.176
	Toceranib	(181, 338)	(0.206, 0.447)	(0.086, 0.292)	(0.086, 0.292)
	Control	215	0.259	0.131	0.065
	Control	(125, 288)	(0.152, 0.378)	(0.055, 0.241)	(0.014, 0.175)
**DFI A**	Toceranib	223	0.313	0.199	0.199
	Toceranib	(182, 338)	(0.177, 0.459)	(0.083, 0.351)	(0.083, 0.351)
	Control	198	0.278	0.183	0.091
	Control	(125, 296)	(0.153, 0.418)	(0.078, 0.323)	(0.019, 0.235)
**OS ITT**	Toceranib	318	0.384	0.163	0.082
	Toceranib	(250, 379)	(0.263, 0.505)	(0.082, 0.268)	(0.024, 0.184)
	Control	242	0.349	0.143	0.078
	Control	(197, 331)	(0.235, 0.466)	(0.070, 0.240)	(0.025, 0.171)

Intervals reported are 95% confidence intervals.

ITT = Intent to treat; A = Adherence

(N = 63 toceranib-treated dogs, 63 control dogs)

**Table 3:** Hazard Ratios (HRs) and confidence intervals from Cox regression analysis.

		Unadjusted			Adjusted^a^		
		HR	95% CI	p-value	HR	95% CI	p-value
**DFI**	**ITT**	0.80	(0.54, 1.19)	0.274	0.80	(0.53, 1.20)	0.461
	**Adherence**	0.79	(0.50, 1.23)	0.300	0.76	(0.48, 1.21)	0.252
**OS** ^b^	**1 year**	1.01	(0.66, 1.54)	0.963	1.01	(0.64, 1.59)	0.961
	**2 years**	1.40	(0.72, 2.75)	0.325	1.45	(0.70, 3.00)	0.316

^**a**^Adjusted for weight and gender (male, female).

^b^Proportional hazards violated (p = 0.08 for OS ITT analysis, p<0.05 for all others).

ITT = Intent to treat

**Table 4:** Median disease free interval, median survival (in days) and Cox regression analysis of cancer location.

Analysis	Location	Median (days)	95% CI	HR	95% CI	p-value
**DFI ITT**	**Proximal Humerus**	182	(127, 261)	1.28	(0.82, 2.01)	0.281
	**Other**	233	(184, 296)	-	-	-
**DFI A**	**Proximal Humerus**	182	(124, 338)	1.17	(0.70, 1.94)	0.557
	**Other**	224	(183, 309)	-	-	-
**OS ITT**	**Proximal Humerus**	243	(176, 309)	1.27	(0.82, 1.97)	0.280
	**Other**	318	(242, 352)	-	-	-

(N = 31 proximal humerus, 95 other locations)

ITT = Intent to treat; A = Adherence

### Adverse Events

Adverse events were similar to those previously reported for carboplatin and toceranib. Grade 1 (n = 77) and 2 (n = 18) neutropenia and grade 1 thrombocytopenia (n = 26) were the most common hematologic adverse events observed during carboplatin chemotherapy. Grade 1 diarrhea (n = 31), lethargy (n = 22) and vomiting (n = 18) were also commonly reported during carboplatin administration. The primary toxicities observed during oral therapy were gastrointestinal and hematologic. S1 Table shows adverse event rates for all events common enough to be modeled using Poisson regression. Patients randomized to toceranib experienced more episodes of diarrhea, neutropenia, and weight loss than patients randomized to control, although these toxicities were low-grade and typically resolved with supportive care and dose modifications. Temporary drug discontinuation due to adverse events was usually 1 week in duration. Any dog requiring drug discontinuation longer than 2 weeks was removed from the study. More toceranib-treated dogs (n = 8) were removed from the study for therapy-associated adverse events compared to control dogs (n = 1). Seven dogs developed cyclophosphamide-induced cystitis (n = 2 control; n = 5 toceranib-treated), resulting in replacement of cyclophosphamide with chlorambucil. Gastrointestinal adverse events in one toceranib-treated dog resulted in a switch from cyclophosphamide to chlorambucil.

#### Gastrointestinal

Thirteen dogs experienced grade 1 vomiting while on toceranib, two of which experienced more than one episode. One toceranib-treated dog developed grade 4 vomiting and diarrhea in conjunction with pancreatitis. The vomiting and diarrhea resolved with supportive care and temporary drug discontinuation. Ten control dogs experienced grade 1 vomiting, with 3 dogs experiencing more than one episode. Seventeen toceranib-treated dogs developed grade 1 diarrhea, with 12 dogs experiencing more than 1 episode. Sixteen dogs developed grade 2 diarrhea while on toceranib, with 6 dogs experiencing more than 1 episode. Two toceranib-treated dogs developed grade 3 diarrhea, with one dog experiencing 3 episodes of diarrhea. Both dogs with grade 3 diarrhea were removed from the study due to gastrointestinal adverse events. Seven dogs not receiving toceranib developed grade 1 diarrhea, with one dog experiencing 2 episodes of diarrhea. Anorexia was generally mild and limited to grade 1 and 2 adverse events in both arms of the study.

#### Hematologic

Grade 1 neutropenia was the most common hematologic adverse event in toceranib-treated dogs, with 14 dogs developing transient neutropenia. No dose adjustments or temporary drug discontinuation were necessary due to transient low-grade hematologic toxicity. Six dogs receiving toceranib, and 8 control dogs experienced grade 1 thrombocytopenia. One dog experienced a grade 5 thrombocytopenia which occurred in the setting of widespread metastatic disease.

#### Biochemical

The majority of elevations in liver transaminases were grade 1 and 2. Two dogs receiving toceranib experienced grade 3 ALT elevations that resolved with a temporary toceranib discontinuation and supportive care. One control dog experienced a grade 3 and 4 ALT and ALP elevation, respectively, which resolved with temporary discontinuation of oral therapy.

#### Neuromuscular

Grade 1 and 2 weakness was seen in 4 toceranib-treated dogs and 3 control dogs. Two dogs receiving toceranib developed grade 3 weakness, with three separate episodes occurring in 1 dog. In both dogs, each episode resolved with a temporary toceranib discontinuation. Low-grade musculoskeletal pain/lameness was noted in the absence of progressive disease in 8 toceranib-treated dogs and 5 control dogs.

#### Other

Other adverse events observed in dogs enrolled in this study included: elevated creatinine kinase; otitis media; fever; urinary tract infection; epistaxis; stranguria; hyperglycemia; hypoglycemia; indolent ocular ulcer; seizure; shaking/panting; skin erythema; pyoderma; hyperkalemia; hypokalemia; hypoalbuminemia; hypercalcemia; ataxia; motor neuropathy; sensory neuropathy. These adverse events were believed to be unrelated to toceranib/oral therapy, and instead likely represent progression of disease or other co-morbid conditions.

## Discussion

The benefits of amputation and adjuvant chemotherapy for dogs with OSA are well established in veterinary medicine, however most dogs will succumb to metastatic disease within 1 year of amputation and adjuvant chemotherapy, with 2 year survival rates reported in most studies of only 10–15% [8,46]. Despite numerous attempts to improve outcome, little progress has been made in extending survival for affected dogs. The purpose of this clinical trial was to determine whether the addition of metronomic chemotherapy using piroxicam and cyclophosphamide alone or in combination with toceranib would impact both disease free interval and overall survival in dogs with appendicular OSA following amputation and carboplatin chemotherapy. Results from this study demonstrate that piroxicam and cyclophosphamide metronomic therapy with toceranib did not improve median DFI, median OS or the 1-year survival rate in dogs with appendicular OSA over piroxicam and cyclophosphamide alone.

The median DFI and survival times reported here are similar to those reported for dogs that receive carboplatin chemotherapy after amputation [3,7]. Furthermore, the 1-year survival rates of 38.4% and 34.9% for toceranib-treated dogs and control dogs, respectively, are comparable to the 35.4% 1-year survival rate reported for carboplatin alone. Analogous results have been obtained with doxorubicin alone or in combination with carboplatin, cisplatin alone, and carboplatin given concurrently with gemcitabine [5,8,47–49]. A recent retrospective study compared multiple chemotherapy protocols used for canine appendicular OSA post-amputation. Differences in dose intensity and the chemotherapy protocol chosen did not significantly influence the reported median DFI and OS of 291 days and 284 days, respectively [8]. These data support the notion that alteration of therapeutic approaches to treat microscopic metastatic disease using currently available cytotoxic chemotherapeutics is unlikely to result in significant benefit for dogs with OSA.

Therapeutic approaches that target both the tumor itself as well as the tumor microenvironment are increasingly recognized as important components in regulating the metastatic process and several studies have investigated this approach. For example, studies in veterinary medicine have reported on safety and efficacy of metronomic chemotherapy protocols in the treatment of various tumors [9,10,50,51]. The combination of metronomic cyclophosphamide (10 mg/m2) and piroxicam (0.3 mg/kg) was shown to be well-tolerated and suggests that metronomic chemotherapy prolongs DFI for incompletely resected canine soft tissue sarcomas [9]. The use of metronomic cyclophosphamide has been also shown to decrease the number of circulating regulatory T-cells (Treg) and tumor microvessel density in tumor-bearing dogs when cyclophosphamide was administered at 15 mg/m2/day [11]. Unfortunately, the doses and regimens used in metronomic chemotherapy protocols are largely anecdotal, and predictive biomarkers of clinical benefit in dogs with OSA have not been thoroughly evaluated. In the present study cyclophosphamide and piroxicam were dosed at 10 mg/m2 and 0.3 mg/kg EOD, respectively. This therapeutic regimen was established based on the available literature at the beginning of the study, prior to demonstration of the immunomodulatory and antiangiogenic effects of higher doses of cyclophosphamide. Given the reported immunomodulatory and anti-angiogenic effects of cyclophosphamide used at higher doses, it is possible that the lower dose of cyclophosphamide in the present study and the use of EOD dosing resulted in a treatment regimen insufficient to significantly affect the tumor microenvironment, the number of circulating Tregs, or both.

While biologic activity of toceranib in metastatic canine appendicular osteosarcoma has been reported [37], the lack of improvement in DFI and OS in the present study suggests there may be different molecular drivers at different stages of disease in canine OSA. It is possible that while effects on tumor growth are observed in dogs with macroscopic metastatic pulmonary lesions following toceranib therapy, microscopic lesions may become resistant to therapy with in a short period of time, thereby negating any potential therapeutic value. Data generated from several murine models have suggested that VEGF inhibitors modulate the tumor microenvironment in a manner that can actually accelerate metastatic tumor growth [28,29,52]. There has therefore been concern that the use of VEGFR inhibitors in the setting of microscopic disease could actually promote a more aggressive phenotype. Importantly, this study demonstrated that the use of toceranib in dogs with microscopic metastatic OSA did not result in shortened survival times or aberrant patterns of metastasis that would indicate the induction of a more aggressive disease phenotype following treatment.

Recently, administration of toceranib at 2.75 mg/kg EOD to cancer-bearing dogs was associated with a significant decrease in circulating Tregs, possibly through indirect immunomodulatory mechanisms [39]. Doses of toceranib below the label dose (ranging from 2.4–2.9 mg/kg EOD) still result in target inhibition, supporting the use of 2.75 mg/kg in the present study [37]. Therefore, the lack of improvement in DFI and OS reported here is unlikely secondary to the use of a lower dose of toceranib than the current label dose. Although VEGFR inhibition was not specifically assessed in this study, upregulation of plasma VEGF concentrations following toceranib treatment has been previously demonstrated in dogs receiving 2.4–2.9 mg/kg EOD, consistent with effective VEGFR2 inhibition [37].

Adverse events necessitating administration of chlorambucil in place of cyclophosphamide occurred in 8 dogs, with all but one of these due to sterile cystitis. The overall incidence of cyclophosphamide-induced cystitis is low in the veterinary literature, and increased risk has been associated with a higher cumulative cyclophosphamide dose and higher dose intensity [53]. As there were a small number of dogs in each group that developed cystitis (n = 2 control, n = 5 toceranib), and it is likely coincidence that a higher number of toceranib-treated dogs developed cystitis. The use of metronomic chlorambucil has been associated with some antitumor activity in a variety of spontaneous canine tumors, and decreases in numbers of circulating Tregs compared to baseline has been documented in dogs receiving chlorambucil [51]. While the immunomodulatory effects of metronomic cyclophosphamide and chlorambucil have been reported, the impact of chlorambucil administration in this study is not known. However, given the small number of dogs that went on to receive this drug in place of cyclophosphamide, it was unlikely to significantly impact outcome in either group.

Multiple investigations have reported on the prognostic significance of OSA occurring in the proximal humerus [3,7,54–57]. While decreased survival times were noted in dogs with tumors located in the proximal humerus in the current study, the difference in OS was not significant compared to other tumor locations. This may have been due to the small number of dogs that enrolled in the study with proximal humerus disease (n = 31). Alternatively, it is possible that the metronomic therapy had an effect on outcome in this population to improve survival. Future controlled studies would be necessary to determine the benefits of metronomic chemotherapy in this subset of patients.

Toceranib-treated dogs had a higher adverse event profile compared to control dogs, although the adverse events reported were consistent with those expected based on prior published studies. It is important to note that in the current study, dogs experiencing adverse events while on toceranib were effectively managed with drug holidays, concomitant medications and/or dose reductions. The frequency of adverse events was lower than that reported in a recent clinical trial of dogs with various solid tumors receiving toceranib at doses of 2.4–2.9 mg/kg [37]. In this study, the adverse event profile was far superior to that associated with the label dose of toceranib (3.25 mg/kg) but both adequate drug exposure and biologic activity were maintained [37]. Despite the inherent difficulties in comparing findings from various studies, the dose of toceranib utilized in the present study (2.75 mg/kg) was better tolerated compared to the label dose, allowing improved adherence to the treatment protocol [36,37].

While necropsy was not required in the current study and the detection of metastatic disease was largely dependent on clinical findings and thoracic radiographs, it is unlikely that gross metastatic disease in other locations was missed in many dogs. The incidence of gross OSA metastasis detectable with abdominal ultrasound has been previously reported, with abdominal metastasis identified in 0–1.7% of dogs that do not have evidence of pulmonary metastatic disease [58,59]. Additionally, the reported rate for detecting unsuspected osseous metastasis with nuclear scintigraphy in dogs with appendicular OSA at the time of initial presentation (prior to amputation) is 7.8% [60]. Thus, while the lack of necropsy may have contributed to an under-reporting of the true incidence of metastasis, thoracic radiography is considered a reliable diagnostic procedure to detect metastatic disease for most dogs following amputation and chemotherapy, with extrathoracic metastases usually identified by virtue of clinical signs (i.e., lameness for bone metastasis).

It is possible that the use of compounded therapeutics could have impacted our results. The Apothecary Shoppe (now Avella Specialty Pharmacy) was chosen as the supplier of compounded cyclophosphamide and piroxicam as this pharmacy is certified by the Pharmacy Compounding Accreditation Board. However it is recognized that compounding may have resulted in inadvertent overdosing or under dosing, resulting in increased toxicity or sub-therapeutic drug concentrations in some patients, respectively.

A potential weakness of the current study is that the metronomic treatment regimen began after completion of carboplatin chemotherapy. This may have given the remaining microscopic metastatic tumor cells time to develop resistance to multiple therapies, thus negating any impact of the subsequent treatment. While the use of metronomic cyclophosphamide and piroxicam administered concurrently with carboplatin for dogs with appendicular osteosarcoma was reported to be well-tolerated in another study [50], no improvement in survival time was noted with concurrent administration. Nevertheless, it may be warranted to determine if concurrent use of toceranib in combination with metronomic chemotherapy and carboplatin significantly impacts DFI and OS.

## Conclusions

The incorporation of toceranib into a commonly employed metronomic chemotherapy protocol failed to improve the DFI, OS and 1-year survival rates in dogs with appendicular OSA after amputation and carboplatin chemotherapy compared to metronomic therapy alone. Furthermore, the DFI, OS and 1-year survival rates in this study were similar to those previously found when dogs with OSA undergo amputation and carboplatin chemotherapy treatment alone. This study underscores the need for thorough interrogation of the molecular mechanisms that drive tumor progression at various stages of disease so that novel approaches aimed at optimizing activity against the tumor microenvironment can be successfully employed.

## Supporting Information

S1 Table Adverse Event Rates (number/30 days) and Rate Ratios (RRs). Table only includes events that were experienced by at least five patients and at least two in each treatment group.
(DOCX)
