# Supplementary material for: Impact of Toceranib/Piroxicam/Cyclophosphamide Maintenance Therapy on Outcome of Dogs with Appendicular Osteosarcoma following Amputation and Carboplatin Chemotherapy: A Multi-Institutional Study
Source: PLoS One. 2015 Apr 29;10(4):e0124889. doi: 10.1371/journal.pone.0124889 (PMC4414350; doi:10.1371/journal.pone.0124889)
Supplement: S1 Table — Table only includes events that were experienced by at least five patients and at least two in each treatment group. (DOCX) [file pone.0124889.s001.docx]

**Table S1. Adverse Event Rates (number/30 days) and Rate Ratios (RRs)*.**

|  | **Toceranib** | | | **Control** | | | **Unadjusted RR** | | **Adjusted RR^b^** | |
| --- | --- | --- | --- | --- | --- | --- | --- | --- | --- | --- |
|  | **N^a^** | **Rate** | **95% CI** | **N^a^** | **Rate** | **95% CI** | **RR** | **95% CI** | **RR** | **95% CI** |
| Vomiting | 15 | 0.101 | (0.062, 0.165) | 6 | 0.057 | (0.026, 0.125) | 1.77 | (0.70, 4.48) | 1.51 | (0.61, 3.73) |
| Diarrhea | 23 | 0.308 | (0.227, 0.419) | 8 | 0.051 | (0.027, 0.097) | 6.01 | (2.96, 12.22) ^††^ | 4.96 | (2.51, 9.80) ^††^ |
| Lethargy | 7 | 0.051 | (0.023, 0.111) | 3 | 0.017 | (0.006, 0.052) | 2.96 | (0.76, 11.56) | 1.80 | (0.59, 0.72) |
| Neutropenia | 15 | 0.106 | (0.069, 0.164) | 3 | 0.017 | (0.006, 0.049) | 6.21 | (2.00, 19.27) ^‡^ | 5.50 | (1.84, 16.42) ^‡^ |
| Weight loss | 12 | 0.066 | (0.040, 0.109) | 2 | 0.011 | (0.003, 0.046) | 5.77 | (1.31, 25.00) ^†^ | 5.17 | (1.23, 21.71) ^†^ |
| BUN | 10 | 0.056 | (0.032, 0.098) | 9 | 0.057 | (0.032, 0.102) | 0.98 | (0.43, 2.20) | 0.65 | (0.29, 1.46) |
| ALT | 6 | 0.040 | (0.019, 0.086) | 8 | 0.063 | (0.032, 0.124) | 0.65 | (0.23, 1.78) | 0.68 | (0.26, 1.76) |
| AST | 7 | 0.040 | (0.020, 0.082) | 4 | 0.029 | (0.011, 0.075) | 1.42 | (0.43, 4.67) | 1.02 | (0.32, 3.24) |
| CK | 4 | 0.025 | (0.010, 0.066) | 6 | 0.057 | (0.026, 0.125) | 0.44 | (0.13, 1.54) | 0.37 | (0.11, 1.17) |
| Weakness | 6 | 0.040 | (0.016, 0.101) | 3 | 0.017 | (0.006, 0.052) | 2.37 | (0.56, 9.94) | 0.90^c^ | (0.41, 8.69) |
| Thrombocytopenia | 6 | 0.035 | (0.016, 0.077) | 10 | 0.068 | (0.038, 0.123) | 0.52 | (0.20, 1.37) | 0.70 | (0.26, 1.88) |
| Pain | 5 | 0.035 | (0.015, 0.085) | 3 | 0.017 | (0.006, 0.049) | 2.07 | (0.52, 8.22) | 1.68 | (0.40, 7.11) |
| Lameness | 3 | 0.015 | (0.005, 0.044) | 2 | 0.017 | (0.004, 0.075) | 0.89 | (0.14, 5.52) | 0.75 | (0.09, 6.27) |

*Table only includes events that were experienced by at least five patients and at least two in each treatment group.

**^a^**Number of patients with at least one event.

^b^Adjusted for weight and gender (male/female).

^c^Adjusted only for weight due to zero events among males.

^†^p < 0.05

^‡^p < 0.01

^††^p < 0.001
